# Nutritional assessment of critically ill patients: validation of the modified NUTRIC score

**DOI:** 10.1038/s41430-017-0008-7

**Published:** 2017-11-23

**Authors:** Manon CH de Vries, WAC (Kristine) Koekkoek, Marieke H Opdam, Dick van Blokland, Arthur RH van Zanten

**Affiliations:** 1 Resident Intensive Care, Department of Intensive Care Medicine, Gelderse Vallei Hospital, Willy Brandtlaan 10, 6716 Ede, The Netherlands; 2 Resident Internal Medicine, Department of Internal Medicine, Gelderse Vallei Hospital, Willy Brandtlaan 10, 6716 Ede, The Netherlands; 3 Resident Emergency Medicine, Emergency Department, Westfriesgasthuis, Maelsonstraat 3, 1624 NP Hoorn, The Netherlands; 4 ICU RN, Applications Computer Technology Specialist, Department of Intensive Care Medicine, Gelderse Vallei Hospital, Willy Brandtlaan 10, 6716 RP Ede, The Netherlands; 5 Internist-intensivist, Department of Intensive Care Medicine, Medical Hospital Director, Gelderse Vallei Hospital, Willy Brandtlaan 10, 6716 RP Ede, The Netherlands

## Abstract

**Background/objectives:**

In order to identify critically ill patients with high nutritional risk the modified NUTrition Risk in the Critically ill (mNUTRIC)-score was developed. This score aims to identify patients that will benefit from nutritional interventions. Few data are available on its validity. In The Netherlands, the MUST-score, a nutritional assessment tool for non-ICU patients, is commonly used in the ICU. To validate the mNUTRIC-score in Dutch ICU patients and compare its prognostic performance with the MUST-score.

**Subjects/methods:**

A single-center retrospective cohort study among 475 mechanically ventilated patients. Prognostic performance of the mNUTRIC and MUST-scores were assessed and compared for discriminative abilities for 28-day mortality and prolonged mechanical ventilation (>2 days).

**Results:**

The discriminative ability of the mNUTRIC-score for 28-day mortality is (ROC-AUC) 0.768 (95% CI 0.722–0.814) with an associated LR+ of 1.73 (95% CI 1.53–1.95) and LR− of 0.24 (95% CI 0.14–0.39) when comparing low with high (>4) scores. Comparing low with high MUST-scores (>1) a ROC-AUC of 0.513 (95% CI 0.445–0.587) and LR+ of 1.05 (95%CI 0.77–1.45) and LR− of 0.97 (95% CI 0.71–1.17) for mortality were found.

The discriminative ability for prolonged ventilation was 0.666 (95% CI 0.616–0.716) and 0.532 (95% CI 0.469–0.594) for the mNUTRIC and MUST-scores, respectively.

**Conclusions:**

The prognostic performance of the mNUTRIC-score for 28-day mortality is fair and comparable to other validation studies. The association with prolonged ventilation was not confirmed by our results. The mNUTRIC-score has better performance than the commonly used MUST-score. Therefore, we suggest abandoning use of the MUST-score and to recommend introduction of the mNUTRIC-score for the nutritional risk assessment of critically ill patients.

## Introduction

Malnutrition in critically ill patients is associated with poor outcomes, including impaired wound healing, higher rates of nosocomial infections, and all-cause mortality [1, 2]. Nutritional status of patients admitted to the intensive care unit (ICU) is influenced by both chronic and acute starvation, but also by the severity of the underlying pathophysiological processes leading to ICU admission. This typically induces a marked catabolic response leading to rapid loss of lean body mass, varying from 5% in single-organ failure to 25% in multi organ dysfunction syndrome (MODS), during the first 10 days after ICU admission [3, 4].

Nutritional therapy can improve outcomes associated with malnutrition in critically ill patients [5]. To identify ICU patients most likely to benefit from nutritional support, validated tools are required. Recently, Heyland et al. [6] published the first nutritional risk assessment tool specifically designed for critically ill patients: the nutrition risk in the critically Ill score (NUTRIC-score), Table 1. This score aims to identify critically ill patients that benefit from aggressive protein-energy provision during ICU stay, thereby improving mortality rates and ventilation duration. During the development of the NUTRIC-score the effects of nutritional interventions for ICU patients with specific baseline characteristics were evaluated in order to stratify effects according to baseline risk. The NUTRIC-score combines prehospitalization parameters like chronic (BMI) and acute starvation (prehospital admission duration) with acute (Interleukin-6) and chronic inflammatory parameters (number of comorbidities) and severity of illness (APACHE-II and SOFA-scores) on ICU admission, to assess nutritional risk and associated outcomes (mortality and ventilation duration). The NUTRIC-score was validated and a high score is associated with higher 28-day mortality and longer duration of mechanical ventilation. However, the NUTRIC-score was derived from and validated within the same database, which limits its external validity [6]. Another limitation of the NUTRIC-score is the measurement of interleukin-6 (IL-6), which is not routinely available in most ICUs. Moreover, Heyland et al. [6] have stated that IL-6 only increased the c-index by 0.007 (from 0.776 to 0.783), being neither clinically nor statistically different. They have therefore suggested that in settings where IL-6 is not available this could be dropped from the score. This adjusted score is called the modified NUTRIC score (mNUTRIC). Although the mNUTRIC-score seems easier to implement into practice than the NUTRIC score, it had not been validated extensively.

**Table 1:** The NUTRIC without IL-6

Variable	Range	Points
Age	<50	0
	50 to <75	1
	≥75	2
APACHE II	<15	0
	15 to <20	1
	20 to 28	2
	≥28	3
SOFA	<6	0
	6 to <10	1
	≥10	2
Number of comorbidities	0 to 1	0
	≥2	1
Days from hospital to ICU admission	0 to <1	0
	≥1	1
Sum of points	Category	Explanation
5–9	High score	Associated with worse clinical outcome (mortality, ventilation)
0–4	Low score	The patients have a low malnutrition risk

NUTRIC-score with strata for low and high risk as described by Heyland et al. [6] on the Canadian Critical Care Nutrition Practice Guidelines website: www.criticalcarenutrition.com

As identification of critically ill patients with high nutritional risk is important to reduce morbidity and mortality the need for an easy to implement, low cost, highly effective score is undeniable. In spite of the fact that the NUTRIC score seems effective, inclusion of the costly IL-6 measurement makes it unattractive for widespread implementation. Therefore, the mNUTRIC seems to be the most promising nutritional risk assessment tool and further validation is warranted.

In addition, another nutritional risk assessment tool, the Malnutrition Universal Screening Tool (MUST) score, is commonly used for hospitalized patients, but has shown limited performance in critically ill patients [7–9]. However, because in The Netherlands the MUST-score has been used for years as a quality indicator to benchmark hospitals and until recently no other tools were available to assess nutritional risk in ICU patients, the MUST-score has been frequently used in critically ill patients [10].

Our main objective was to validate the modified NUTRIC-score (mNUTRIC) in a Dutch ICU population, reflected by the impact on mortality and whether patients received proglonged mechanical ventilation.

Second, we performed a subgroup analysis of patients of which the MUST-score was available and compared the performance of the mNUTRIC-score to the MUST-score, since the MUST-score is still frequently used in ICU patients in The Netherlands.

## Materials and methods

For this single-center cohort study, we retrospectively collected data from all patients fulfilling the inclusion criteria, who were admitted to the ICU of our University-affiliated teaching hospital between 1 July 2011 and 30 June 2013. Inclusion criteria were: adult critically ill patients (≥18 years), requiring (non)-invasive mechanical ventilation within 24 h after admission. Patients were excluded if the time between ICU admission and discharge was less than 24 h, if data on mNUTRIC variables were incomplete, or in case of pregnancy. Readmitted patients from the ward to the ICU within the same hospital admission were not eligible.

### Ethical approval

The institutional review board of Gelderse Vallei Hospital approved the study and waived informed consent for reasons of the retrospective design, large number of included patients and anonymization of patient identifiers before analysis.

### Data collection

Data extraction were performed automatically using SAS Enterprise Guide queries (version 7.12HF1), from the MetaVision (Patient Data Management System MetaVision, iMDsoft Tel Aviv, Israel) database and other hospital electronic patient records. Baseline characteristics were listed and selected at calculating mNUTRIC- and MUST-scores; age at admission, gender, primary admission diagnosis, admission type (medical, elective, and non-elective surgery), comorbidities, APACHE-II score, SOFA-score, duration in hospital prior to ICU admission, BMI, unplanned weight loss in past 3–6 months, nutritional intake in the 5 days prior to ICU admission.

Mortality data were collected up to 28 days after ICU discharge from hospital records, including records from hospital admissions and visits to outpatient clinics.

### Calculation of mNUTRIC-score

We used the modified 9 points scale of the NUTRIC-score, the mNUTRIC-score (Table 1). Based on this adapted version, we defined the cutoff points 0–4 as “low scores”, with low risk and the cutoff points 5–9 as “high scores”, with associated worse clinical outcomes regarding mortality and mechanical ventilation (Table 1) [6].

### Calculation of the MUST-score

The MUST-score comprises the following variables: BMI, unplanned weight loss in the 3–6 months prior to admission, acute illness and nutritional intake in the days prior to admission (Table 2) [7]. We considered MUST-scores >1 as high nutritional risk.

**Table 2:** MUST score

Variable		Points
BMI (kg/m^2^)	>20 (>30 Obese)	0
	18.5–20	1
	<18.5	2
Unplanned weight loss in past 3–6 months	<5%	0
	5–10%	1
	>10%	2
Acute disease effect score	If patient is acutely ill and there has been or is likely to be no nutritional intake for >5 days	2
	If not	0
Sum of points	Category	Explanation
0	Low Risk	Routine clinical care
1	Medium risk	Observe
2 or more	High risk	Treat

MUST-score with strata for nutritional risk referenced to the original article and adapted from the BAPEN website [8]

### Study end points

The primary aim was to validate the mNUTRIC-score with respect to 28-day mortality and prolonged mechanical ventilation (>2 days). Second, a subgroup analysis to address the performance of mNUTRIC-score compared with the MUST-score regarding 28-day mortality and proglonged mechanical ventilation was performed.

### Data analysis

Descriptive data are reported as frequency and percentage when appropriate. In case of skewed distribution data are shown as median and inter quartile range (IQR).

### Statistical analysis

We defined patients alive after 28 days post ICU admission as survivors; patients who died within 28 days were considered non-survivors.

Baseline characteristic differences between the groups’ survivors and non-survivors for categorical variables were tested with *χ* ^2^ tests. Continuous variables were tested with Wilcoxon rank-sum tests (Mann–Whitney test).

The primary endpoint was assessed by calculation of sensitivity, specificity, positive, and negative predictive values. The proportion of non-survivors or patients with a ventilation duration of more than 2 days, that was correctly identified by the mNUTRIC-score (>4 points), was defined as sensitivity. The proportion of survivors or patients with less than 2 days of mechanical ventilation, that was correctly identified by the mNUTRIC-score (≤4 points), was defined as specificity.

Sensitivity and 1-specificity was used to plot receiver operating characteristic curves (ROC-curves) in which the area under this curve (AUC) represents the discriminative ability of the mNUTRIC-score screening tool, as a binary function for mortality and 2 days ventilation. To value the prediction of prolonged mechanical ventilation we used 2 days as a cutoff, to construct an ROC-curve. Two days was arbitrarily selected as cutoff as nutritional interventions are considered beneficial to patients with prolonged duration of ventilation. We considered an AUC of 0.90–1.00 as excellent, 0.80–0.90 as good, 0.70–0.80 as fair, 0.60–0.70 as poor and 0.50–0.60 as fail.

Additionally, positive and negative likelihood ratios were calculated to quantify the association between high mNUTRIC-scores (>4 points) and 28-day mortality.

To compare the performance of the mNUTRIC-score with the MUST-score in subgroup analysis, sensitivity, specificity, positive, and negative predictive value as well as ROC-curves and likelihood ratios were constructed in the same manner regarding the MUST-score.

Finally, to compare our results regarding the performance of the mNUTRIC-score for mortality to the initial validation cohort of Heyland et al. and the predicted mortality rate of the mNUTRIC-score an histogram was constructed.

IBM SPSS statistics for Windows version 19.0 (IBM Corporation, released 2010, Armonk, New York, USA), was used for statistical analysis. We considered *p* < 0.05 to be statistically significant.

## Results

### Patients

**Fig. 1:**
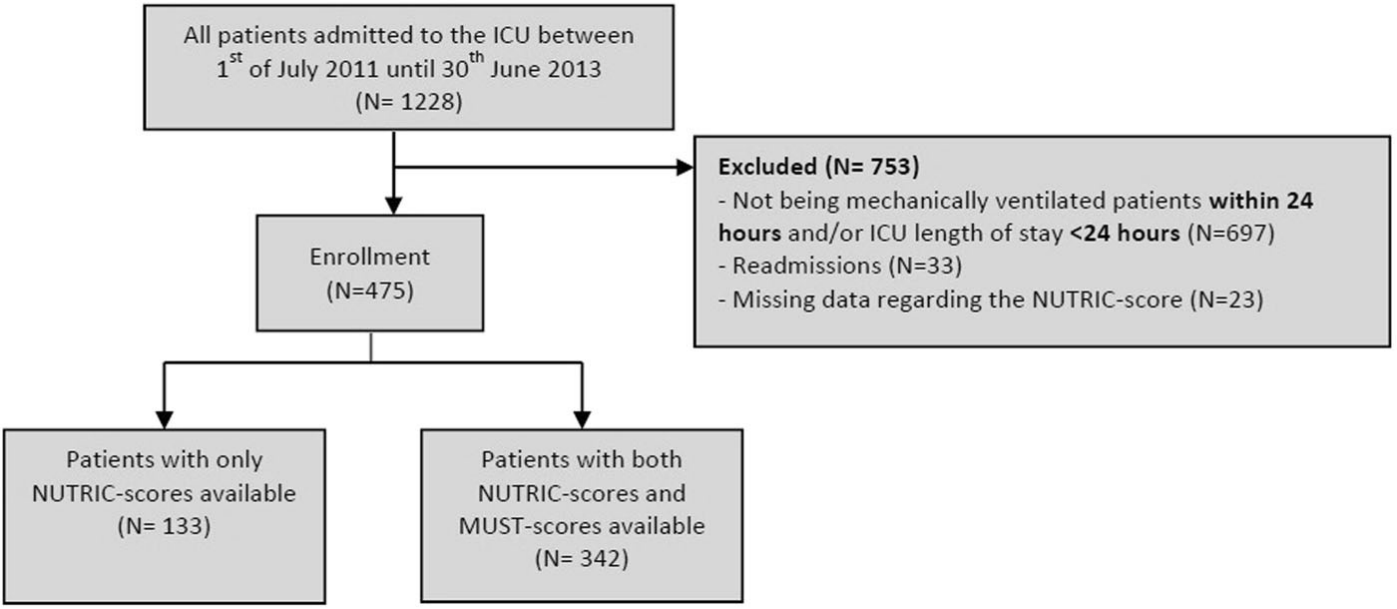
Flowchart of validation cohort

During the study period 1228 patients were admitted to our ICU, all were eligible for inclusion. We excluded 753 patients. Reasons for exclusion were delayed intubation and/or short ICU length of stay (*N* = 697), ICU readmission (*N* = 33), and insufficient data regarding mNUTRIC-score parameters (*N* = 23), (Fig. 1). A total of 475 patients were enrolled.

Baseline characteristics are shown in Table 3. Significant differences were observed between survivors and non-survivors regarding age, APACHE-II-score, SOFA-score, BMI, number of comorbidities, admission category, and median mNUTRIC-score.

**Table 3:** Baseline patient characteristics

	Non-survivors (*n* = 121)	Survivors (*n* = 354)	*P* value
Age (years)	75 (67.50–81.00)	68 (57.00–76.00)	<0.001
Gender: female	56 (46.3%)	150 (42.4%)	0.454
Male	65 (53.7%)	204 (57.6%)	
APACHE II-score (points; 0–72)	26 (20–31)	18 (15–24)	<0.001
SOFA-score (points; 0–24)	9 (7–10)	7 (5–9)	<0.001
Duration in hospital prior to ICU admission (days)	0.71 (0.05–3.81)	1.02 (0.10–3.81)	0.291
BMI (kg/m^2^)	25.6 (22.90–28.40)	26.5 (23.98–29.40)	0.011
Number of comorbidities			0.029
0, 1	17 (14.0%)	83 (23.4%)	
2, 3, 4+	104 (86.0%)	271 (76.6%)	
Admission category			<0.001
Surgical	22 (18.2%)	151 (42.7%)	
Medical	99 (81.8%)	203 (57.3%)	
Median NUTRIC-score (0–9)	6 (5–7)	5 (3–6)	<0.001
Number of MUST-score	89	253	
Available	0.9 (0–2)	0.75 (0–1)	0.605
Median MUST score (0–6)			
Primary admission diagnosis			0.310
Cardiovascular/vascular	35 (28.9%)	96 (27.1%)	
Respiratory	37 (30.6%)	105 (29.7%)	
Gastrointestinal	22 (18.2%)	71 (20.1%)	
Neurologic	6 (5.0%)	6 (1.7%)	
Sepsis	13 (10.7%)	45 (12.7%)	
Trauma	1 (0.8%)	2 (0.6%)	
Metabolic	4 (3.3%)	6 (1.7%)	
Post-operative conditions	0 (0.0%)	14 (4.0%)	
Renal	2 (1.7%)	7 (2.0%)	
Orthopedic	1 (0.8%)	2 (0.6%)	

Data are presented as median with inter quartile range (IQR) analyzed with Mann–Whitney tests or number (N) with percentage (%) analyzed with *χ* ^2^-tests

*APACHE II* acute physiology and chronic health evaluation II, *BMI* body mass index, *MUST* malnutrition universal screening tool, *NUTRIC-score* nutrition risk in the critically Ill score, *SOFA-score* sequential organ failure assessment score

### Primary outcome

**Fig. 2:**
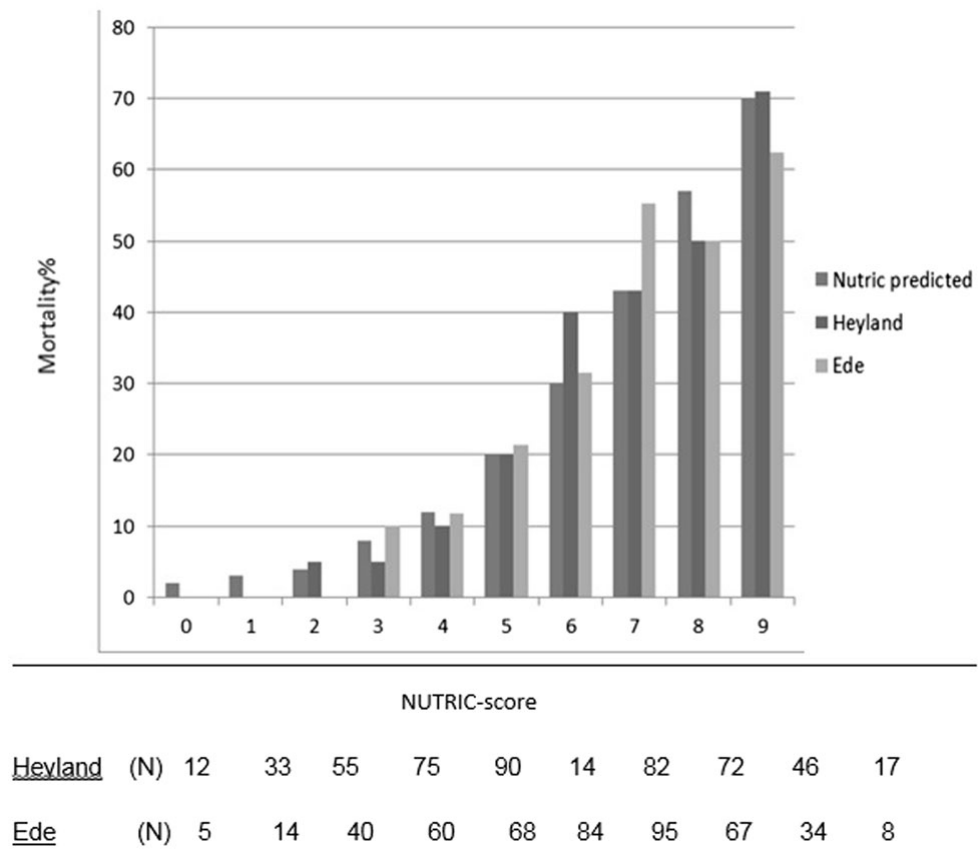
NUTRIC-scores and 28 days mortality. NUTRIC predicted, first bar, is a predicted value, no patients. Heyland et al. [6], middle bar, with number of patient (N), Ede, last bar, number of patients (N) per NUTRIC score category

In total 121 patients (25.5%) died within 28 days after ICU admission (Table 4). The mNUTRIC-score shows a sensitivity of 88.4% and specificity of 48.9%, with a positive and negative predictive value of 37.2% and 92.5%, respectively, (Table 4). A high mNUTRIC-score (>4 points) was significantly associated with increased 28-day mortality risk (LR+ 1.73, 95% CI 1.53–1.95; LR− 0.24, 95% CI 0.14–0.39)). The overall discriminate ability of the mNUTRIC-score for predicting 28-day mortality was 0.768 (95% CI 0.722–0.814). Figure 2 compares the distribution of the NUTRIC-score in the initial validation database of Heyland et al. [6] with the distribution of the mNUTRIC-score found in our database and shows predicted and reported 28-day absolute mortality rates per mNUTRIC-point.

**Table 4:** Results from receiver operator curves (ROC)

	AUC	95% CI
NUTRIC-score and mortality	0.768	0.722–0.814
MUST-score and mortality	0.513	0.445–0.587
Combined MUST/NUTRIC-score and mortality	0.679	0.618–0.740
NUTRIC-score and >2 days of ventilation	0.666	0.616–0.716
MUST and >2 days of ventilation	0.532	0.469–0.594

*AUC* area under de curve, *CI* confidence interval

**Fig. 3:**
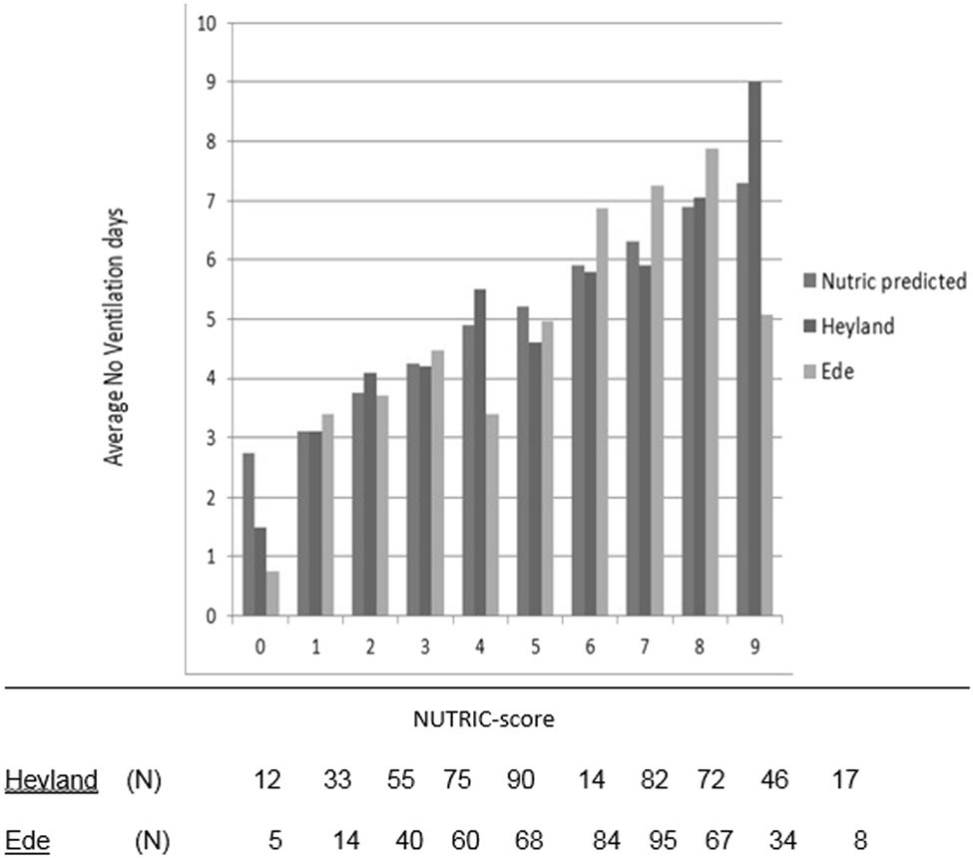
NUTRIC-scores and observed duration of ventilation. NUTRIC predicted, first bar, is a predicted value, no patients. Heyland et al. [6], middle bar, with number of patient (N), Ede, last bar, number of patients (N) per NUTRIC score category

Median duration of ventilation was significantly increased in patients with high mNUTRIC-scores (+2.5 days, *p* < 0.001). An AUC of 0.666 (95% CI: 0.616–0.716) to predict prolonged mechanical ventilation (>2 days) was found. (Fig. 3)

### Secondary outcomes

Of the 475 enrolled patients, MUST-scores could be calculated in 342 cases. There were no differences in baseline characteristics for patients with or without MUST-scores (Supplementary material Appendix 1). The MUST-score was not significantly associated with mortality (LR+1.05, 95% CI 0.77–1.45; LR− 0.97, 95% CI 0.81–1.17; Table 5). The ROC-curve for mortality showed an AUC of 0.513 (95% CI 0.445–0.587). An AUC of 0.532 (95% CI: 0.469–0.594) to predict prolonged mechanical ventilation was found.

**Table 5:** Combined NUTRIC-score and MUST-score vs. Mortality

	Non-survivors	Survivors	Total
Low MUST-score, low NUTRIC-score	9	81	90
High MUST-score, low NUTRIC-score	2	40	42
Low MUST-score, high NUTRIC-score	47	83	130
High MUST-score, high NUTRIC-score	31	49	80
Total	89	253	342

Cutoff point for high MUST-score is >1. Cutoff point for high NUTRIC-score is >4

## Discussion

In this cohort, a fair predictive performance of the mNUTRIC-score was found regarding 28-day mortality based on discriminative abilities (AUC 0.768; 95% CI 0.722–0.814). These results are in line with the initial validation study by Heyland et al. (AUC 0.783) [6] and recently published validation studies of the mNUTRIC-score by Rahman et al. (AUC 0.648) [11] and Mukhopadhyay et al. (AUC 0.71) [12], in Caucasian and Asian populations. In addition to an association between the mNUTRIC-score and 28-day mortality, these studies reported high nutritional adequacy to be associated with a reduction of 28-day mortality in patients with high mNUTRIC-scores (>4) [6, 11, 12]. Because feeding parameters were not available in our cohort, nutritional adequacy could not be analyzed. Therefore, the association between nutritional adequacy, mNUTRIC-scores and mortality could not be confirmed by our results.

The initial validation study by Heyland et al. [6] shows an association of the NUTRIC-score with ventilation duration. However, in our cohort, poor discrimination of the mNUTRIC-score was found with respect to ventilation duration using 2 days as cutoff (AUC 0.666; 95% CI 0.616–0.716).

### Secondary end points

We compared the predictive performance of the mNUTRIC-score to performance of the MUST-score. Although not validated in the ICU setting, the MUST-score is commonly used in Dutch ICUs [10]. Based on discriminative abilities the mNUTRIC-score is superior to the MUST-score in prediction of 28-day mortality in this study. No previous study has compared these nutritional risk assessment tools in the ICU setting. As the MUST-score is not validated in the ICU setting, the association between nutritional adequacy, mortality and the MUST-score has not been assessed and this study suggests inferiority to the mNUTRIC-score, the MUST-score cannot be recommended for nutritional risk assessment in the ICU setting.

### Strengths and limitations

This is the first validation of the mNUTRIC-score in an exclusive European population also analyzing and comparing this score with the commonly used MUST-score in the ICU setting.

Limitations of our study are mainly related to its retrospective design potentially introducing bias and resulting into a limited number of patients included in the groups with the highest and lowest mNUTRIC-scores. Additionally, validation of the mNUTRIC-score in this study is solely based on its discriminative ability. Overall performance and calibration were not statistically tested. Furthermore, we were not able to assess the effects of nutritional adequacy on mortality in patients with different mNUTRIC-scores, because limited nutritional data were available.

### Unanswered questions and further research

Up until now, the mNUTRIC-score and its association with nutritional adequacy and mortality has not been studied prospectively. Observational studies suggest that especially patients with high mNUTRIC-scores may benefit from optimal feeding adequacy during ICU admission, thereby improving survival [6, 11, 12]. Prospective studies are warranted to show the effect of nutritional interventions in critically ill patients according to baseline nutritional risk to study whether outcome can be improved despite a given (nutritional) risk. This should include the effects of baseline nutritional risk and nutritional adequacy on other important outcome measures besides survival, such as muscle mass/strength and the immune response as they remain unclear.

## Conclusions

The discriminative ability of the modified NUTRIC-score for 28-day mortality in Dutch ICU patients is fair and comparable with that found in previous validation studies. However, the association with prolonged ventilation could not be confirmed by our results. The prognostic performance of the MUST-score is less than the mNUTRIC-score. Furthermore, as it is not validated in the ICU population, the MUST-score cannot be recommended for nutritional risk assessment in the ICU. We therefore suggest abandonment of the MUST-score and introduction of the mNUTRIC-score in Dutch ICUs.

## Electronic supplementary material

Suppl Material Appendix I
