# Supplementary material for: Nutritional assessment of critically ill patients: validation of the modified NUTRIC score
Source: Eur J Clin Nutr. 2017 Nov 23;72(3):428–35. doi: 10.1038/s41430-017-0008-7 (PMC5842932; doi:10.1038/s41430-017-0008-7)
Supplement: Supplementary file 1 — Suppl Material Appendix I [file 41430_2017_8_MOESM1_ESM.docx]

| http://eproofing.springer.com/journals_v2/index.php?token=HvoZqx2SczjJkfqmVCWr92EzFBTpUOkDAppendix 1: Baseline characteristics for MUST vs. No MUST scores available. | | | | |
| --- | --- | --- | --- | --- |
|  | **mNUTRIC (n=475)** | **MUST-score available (n=342)** | **No MUST score available (n=133)** | **P-value** |
| Age (years) | 69 (60.00-77.00) | 69.00 (58.75-77.00) | 71.00 (62.50-78.50) | 0.117 |
| Gender: Female  Male | 206 (43.4%)  269 (56.6%) | 145 (42.4%)  197 (57.6%) | 61 (45.9%)  72 (54.1%) | 0.494 |
| Apache II-Score (Points; 0-72) | 20 (16-26) | 20 (16-26) | 20 (16-26) | 0.525 |
| SOFA-score (Points; 0-24) | 7 (5-9) | 7 (5-9) | 7 (5-9) | 0.650 |
| Duration in hospital prior to ICU admission (days) | 1.02 (0.10-3.81) | 1.05 (0.09-3.81) | 0.97 (0.10-3.80) | 0.504 |
| BMI (kg/m^2^); | 26.2 (23.50-29.10) | 26.30 (23.50-29.40) | 26.20 (23.20-28.95) | 0.510 |
| Number of Comorbidities  0, 1  2, 3, 4+ | 100 (21.1%)  375 (78.9%) | 75 (21.9%)  267 (78.1%) | 25 (18.8%)  108 (81.2%) | 0.452 |
| Admission category  Surgical  Medical | 173 (36.4%)  302 (63.6%) | 115 (33.6%)  227 (66.4%) | 58 (43.6%)  75 (56.4%) | 0.042 |
| Median NUTRIC score (0-9) | 5 (3-6) | 5 (4-6) | 5 (3-6) | 0.643 |
| Primary admission diagnosis  *Cardiovascular/vascular*  *Respiratory*  *Gastrointestinal*  *Neurologic*  *Sepsis*  *Trauma*  *Metabolic*  *Post-operative conditions*  *Renal*  *Orthopedic* | 131 (27.6%)  142 (29.9%)  93 (19.6%)  12 (2.5%)  58 (12.2%)  3 (0.6%)  10 (2.1%)  14 (2.9%)  9 (1.9%)  3 (0.6%) | 85 (24.9%)  109 (31.9%)  65 (19.0%)  12 (3.5%)  44 (12.9%)  2 (0.6%)  7 (2.0%)  9 (2.6%)  6 (1.8%)  3 (0.9%) | 46 (34.6%)  33 (24.8%)  28 (21.1%)  0 (0.0%)  14 (10.5%)  1 (0.8%)  3 (2.3%)  5 (3.8%)  3 (2.3%)  0 (0.0%) | 0.216 |

Comparison of groups “mNUTRIC and MUST available” and “Only mNUTRIC available”. Data are presented as median with inter quartile range (IQR) analyzed with Mann-Whitney tests or number (N) with percentage (%) analyzed with chi squared tests. APACHE II: Acute Physiology and Chronic Health Evaluation II, SOFA score: Sequential Organ Failure Assessment score, BMI: Body Mass Index, NUTRIC score: Nutrition Risk in the Critically Ill Score. MUST: Malnutrition Universal Screening Tool.
